# Exploring Relations Between Unique Patient Characteristics and Virtual Reality Immersion Level on Anxiety and Pain in Patients Undergoing Venipuncture: Secondary Analysis of a Randomized Control Trial

**DOI:** 10.2196/53196

**Published:** 2024-07-01

**Authors:** Jeffrey I Gold, Krystal M Akbar, Sandra Avila, Nhat H Ngo, Margaret J Klein

**Affiliations:** 1 Departments of Anesthesiology, Pediatrics, and Psychiatry & Behavioral Sciences Keck School of Medicine University of Southern California Los Angeles, CA United States; 2 Department of Anesthesiology Critical Care Medicine The Saban Research Institute Children's Hospital Los Angeles Los Angeles, CA United States; 3 The Saban Research Institute at Children's Hospital Los Angeles Los Angeles, CA United States; 4 Department of Anesthesiology Critical Care Medicine The Biobehavioral Pain Lab The Saban Research Institute, Children's Hospital Los Angeles Los Angeles, CA United States

**Keywords:** pediatrics, virtual reality, VR, immersion, anxiety, pain management, routine medical procedures, venipuncture, secondary data analysis, mediation, moderation, pain, acute pain, pediatric pain, anxiety sensitivity, pain management

## Abstract

**Background:**

Virtual reality (VR) is a well-researched digital intervention that has been used for managing acute pain and anxiety in pediatric patients undergoing various medical procedures. This study focuses on investigating the role of unique patient characteristics and VR immersion level on the effectiveness of VR for managing pediatric pain and anxiety during venipuncture.

**Objective:**

The purpose of this study is to determine how specific patient characteristics and level of immersion during a VR intervention impact anxiety and pain levels for pediatric patients undergoing venipuncture procedures.

**Methods:**

This study is a secondary data analysis of 2 combined, previously published randomized control trials on 252 pediatric patients aged 10-21 years observed at Children’s Hospital Los Angeles from April 12, 2017, to July 24, 2019. One randomized clinical trial was conducted in 3 clinical environments examining peripheral intravenous catheter placement (radiology and an infusion center) and blood draw (phlebotomy). Conditional process analysis was used to conduct moderation and mediation analyses to assess the impact of immersion level during the VR intervention.

**Results:**

Significant moderation was found between the level of immersion and anxiety sensitivity when predicting postprocedural anxiety (*P*=.01). Patients exhibiting the highest anxiety sensitivity within the standard of care yielded a 1.9 (95% CI 0.9-2.8; *P*<.001)-point elevation in postprocedural anxiety relative to individuals with high immersion levels. No other significant factors were found to mediate or moderate the effect of immersion on either postprocedural anxiety or pain.

**Conclusions:**

VR is most effective for patients with higher anxiety sensitivity who report feeling highly immersed. Age, location of the procedure, and gender of the patient were not found to significantly impact VR’s success in managing levels of postprocedural pain or anxiety, suggesting that immersive VR may be a beneficial intervention for a broad pediatric population.

**Trial Registration:**

ClinicalTrials.gov NCT04268901; https://clinicaltrials.gov/study/NCT04268901

## Introduction

The subjective experience of pain results from complex interactions among biological, psychological, and social factors and is largely informed by early life experiences [1]. Thus, what a child remembers about initial painful experiences is a strong predictor of their response to subsequent painful experiences [2-5]. Anxiety has been shown to moderate children’s memory for procedural pain, increasing the likelihood of remembering more pain than they initially reported [6]. In addition to the unique interplay of pain and anxiety associated with routine medical procedures, the literature reflects that pain and anxiety associated with routine medical care can lead to adverse consequences that can affect lifelong health, such as a negative impact on one’s perception of health care, attempts to escape the distressing medical procedure, poor recovery outcomes, avoidance of preventative health care, and the risk for medical trauma [7-9].

Decreasing pain and anxiety during pediatric medical care is critical to ensuring optimal health and health care experiences for individuals across the lifespan. Best practice guidelines for the treatment of procedural pain and anxiety in pediatric populations have historically been cited as a combination of pharmacological and nonpharmacological interventions [10-13]. While pharmacological interventions (eg, sedatives and opioids) have become increasingly common, these analgesics have been linked to higher mortality risk and longer hospital admissions [14]. Additionally, these medication interventions have been associated with high tolerance, dependence, and unfavorable side effects [15,16]. In fact, when a study compared the efficacy of virtual reality (VR) and opioid therapy as pain management tools during thermal pain stimulation, results indicated that there was no significant difference in pain reduction between the 2 treatment groups [17]. This suggests that VR may be just as effective as routine pharmacological interventions in reducing the experience of pain and anxiety while mitigating the adverse impacts of tolerance, dependence, and other unfavorable side effects.

VR is an immersive and interactive computer-generated environment that has gained traction as a pain and anxiety management strategy within the medical field over the past few decades. In the past 5 years alone, studies have cited VR’s success in reducing pain and anxiety during dental procedures [18], cancer-related treatments [19,20], and burn wound care [21]. However, many children and adolescents view venipuncture—a medical procedure in which a needle is used to draw blood from a vein—as one of the most distressing aspects of attending hospital visits [22,23] due to a combination of fear, anxiety, and pain [24]. This finding holds important considerations for children with acute or chronic illnesses, as they are exposed to needle procedures at a much higher rate than similar-aged peers who do not have medical conditions. Further, children may experience these procedures as more distressing than adults due to receptive language, expressive language, and emotional regulation limitations that impact their understanding of the routine nature of medical procedures, their ability to communicate their experiences of pain, and their ability to cope with painful experiences, respectively [25-27].

Extensive research on the use of VR has established it as a safe, feasible, effective, and efficacious intervention for reducing pain and anxiety associated with routine painful medical procedures in pediatric populations compared with standard of care (SOC) [28-31]. Furthermore, pediatric studies have indicated that children report lower pain, distress, and anxiety scores when using VR, an immersive pain management tool, –than when using iPads (Apple Inc), a passive coping tool [32-36]. However, information regarding the impact of specific unique individual characteristics that affect VR’s level of efficacy has been limited to preliminary studies with outdated technologies. For example, researchers have found that a higher perception of feeling present in the VR simulation is associated with better outcomes [37-39], and recent studies that have explored the effects of age and sex characteristics across a range of pediatric specialty clinics have neither found any significant correlations with pain nor anxiety levels [40,41]. This suggests a need for further investigation into the demographic, medical, and psychological variables that may impact VR’s efficacy to further appreciate “who benefits” from VR interventions and why.

This study aims to identify individual factors that impact the degree of VR’s effectiveness in reducing pain and anxiety during routine painful medical venipuncture procedures (phlebotomy and peripheral intravenous catheter [PIVC] placement) in pediatric patients.

## Methods

### Study Design and Population

This study is a secondary data analysis of 2 combined, previously published randomized control trials on 252 pediatric patients aged 10-21 years observed at an urban pediatric academic medical center (Children’s Hospital Los Angeles) from April 12, 2017, to July 24, 2019 [29,30]. Both randomized control trials implemented the same protocol. A total of 3 clinical environments were used to examine PIVC placement (radiology and an infusion center) [30] and blood draw (phlebotomy) [29]. In total, 125 dyads (patient and caregiver) were randomized to receive the VR intervention while 125 received standard of care (SOC). Patients randomized to the VR group played a multisensory (visual and auditory) VR game, BearBlast, where users traveled on a preset path through a colorful and immersive 3D environment filled with animated landscapes, buildings, and clouds, during which the user’s gaze controlled the direction of a cannon fired to knock down teddy bears. The VR game is equipped with a head-tracking system, enabling the player to look around the virtual environment (VE), controlling the game with only the movement of their head (Oculus Gear VR). Patients and participants were English or Spanish-speaking.

### Measures

Caregivers provided demographic information for patients younger than 18 years of age, while patients older than 18 years completed self-reported demographic questionnaires focused on age, gender, race or ethnicity, and relevant medical history.

### Pain

The Faces Pain Scale-Revised (FPS-R) [42] was used to measure patient pain before and during the PIVC procedure, and uses a horizontal series of 6 faces displaying a range of facial expressions, from no pain (0 points) to significant pain (10 points). Patients and caregivers pointed to the face that indicated the patient’s level of pain. Across multiple studies, the FPS-R has been found to be both a reliable and valid measure of patient pain for children between the ages of 4 and 16 years of age [42].

### Anxiety

A visual analog scale (VAS) was provided to patients and caregivers to measure patient anxiety prior to and after the PIVC procedure. The VAS provides the patient with a vertical image of a thermometer that shifts in color from yellow (accompanied by an image of a neutral face) at the bottom to dark red (accompanied by an image of a distressed face) at the top. Patients and caregivers were asked to point to the specific part of the thermometer that rated the patient’s level of anxiety, with the neutral face scoring as 0 points and the distressed face scoring 10 points. Many studies have evaluated the effectiveness of a VAS and concluded that this type of scale is subject to less bias when compared to categorical scales [43].

### Anxiety Sensitivity

The Childhood Anxiety Sensitivity Index (CASI) was used to measure the patient’s anxiety sensitivity, and it is comprised of a 3-point Likert scale that assesses the patient’s belief that their anxiety will result in a negative consequence, such as sickness, embarrassment, or loss of control. On the CASI, 1 indicates no negative consequences, 2 indicates some negative consequences, and 3 indicates a lot of negative consequences (range 18-54) [30]. Studies have found that the CASI has high internal consistency (α=.87) and good test-retest reliability in both clinical (*r*=0.79) and nonclinical (*r*=0.76) samples [44]. Patients in our study who were missing 1 of the 18 items had their total CASI sum imputed by adding the mean of their 17 complete items to their 17-item sum. Patients missing more than 1 item of the CASI were excluded from CASI analyses.

### VR Immersion

Patients in the VR group completed the Gold-Rizzo Immersion and Presence (GRIP) inventory, which is a 16-item measure that asks the patients to indicate their degree of immersion in the game, with 0 indicating no immersion, 1 indicating little immersion, and 2 indicating a lot of immersion. This measure is comprised of 3 domains—sense of involvement, perceived realism of the VR game, and sense of transportation into the experience [30]. The scores on the GRIP inventory range from 0 to 32 points, with higher scores indicating higher levels of immersion. Patients in the SOC group were given a score of 0 for the GRIP immersion score since they never experienced the VR intervention. Patients in the VR group who were missing 1 or 2 of the 16-item GRIP had their missing items imputed with their nonmissing median to create a total 16-item score. These final scores were categorized into no immersion (score=0), low (score 1-19), medium (20-25), and high immersion (>25) based on our sample’s distribution.

### Data Analysis

Patient demographic and preprocedural characteristics were summarized using the median with IQR for continuous variables while frequency and percentage were used for categorical variables. Group differences were tested using the chi-square or Mann-Whitney *U* test.

Conditional process analysis was used for the moderation and mediation analyses [45]. Separate models were run for the proposed mediation and moderation effects of child characteristics (age, gender, procedure location, and anxiety sensitivity) and level of immersion on the outcomes of postprocedural anxiety VAS and pain FPS-R. All models were controlled for preprocedural pain or anxiety.

When pairwise comparisons were analyzed for significant differences, the Bonferroni multiple comparison adjustment was applied and simultaneous 95% CIs were presented. All *P* values were assessed at the α level of .05. Statistical analyses and data visualization were carried out with SAS (version 9.4; SAS Institute) for Windows and SPSS Statistics (version 28.0; IBM).

### Ethical Considerations

All activities and procedures were approved by the local institutional review board at Children’s Hospital Los Angeles (CHLA-15-00549). All patients older than 18 years provided written informed consent. Caregivers of patients younger than 18 years provided patient assent and written informed consent. All activities and procedures were approved by the local institutional review board at Children’s Hospital Los Angeles.

## Results

A total of 250 patients with complete data were included in the current secondary data analysis. Of the participants, 46% (n=115) were female with a median age of 15 (IQR 13-17.3) years and 51.2% (n=128) were Hispanic or Latinx. A total of 56% (n=141) of the participants underwent phlebotomy procedures, while 17.6% (n=44) and 26% (n=65) were recruited from the radiology and infusion departments, respectively. There were no statistically significant differences in these demographic variables between the SOC and VR groups (all *P*>.05; Table 1). Preprocedural pain FPS-R, anxiety VAS scores, and anxiety sensitivity CASI scores did not differ between groups (Table 1).

**Table 1:** Demographics and baseline characteristics.

Demographics and baseline characteristics		Child condition			All (N=250)		*P* value^a^
		SOC^b^ (n=125)	VR^c^ (n=125)				
Female, n (%)		63 (50.40)	52 (41.6)	115 (46)		.16	
**Race or ethnicity, n (%)**							.09
	Hispanic or Latinx	59 (47.2)	69 (55.2)	128 (51.2)			
	White or non-Hispanic	32 (25.6)	18 (14.4)	50 (20)			
	Other	34 (27.2)	38 (30.4)	72 (28.8)			
**Location, n (%)**							.99
	Radiology	22 (17.6)	22 (17.6)	44 (17.6)			
	Infusion	32 (25.6)	33 (26.4)	65(26)			
	Phlebotomy	71 (56.8)	70 (56)	141 (56.4)			
Age, median (IQR)		15.00 (13.0-17.0)	15.00 (13.00-17.99)	15.00 (13-17.31)		.75	
Preprocedural FPS-R^d^ (n=247), median (IQR)		1.00 (0.00-1.00)	1.00 (0.00-1.00)	1.00 (0.00-1.00)		.61	
Preprocedural anxiety VAS^e^ (n=247), median (IQR)		1.51 (0.33-3.41)	1.33 (0.28-3.41)	1.38 (0.32-3.15)		.90	
CASI^f^ score (n=246), median (IQR)		28.0 (24.4-32.0)	27.0 (24.0-32.0)	28.0 (24.0-32.0)		.80	

^a^*P* values from the chi-square test for categorical variables and the Mann-Whitney *U* test for continuous variables.

^b^SOC: Standard of care.

^c^VR: virtual reality.

^d^FPS-R: Faces Pain Scale-Revised.

^e^VAS: visual analog scale.

^f^CASI: Childhood Anxiety Sensitivity Index.

**Figure 1:**
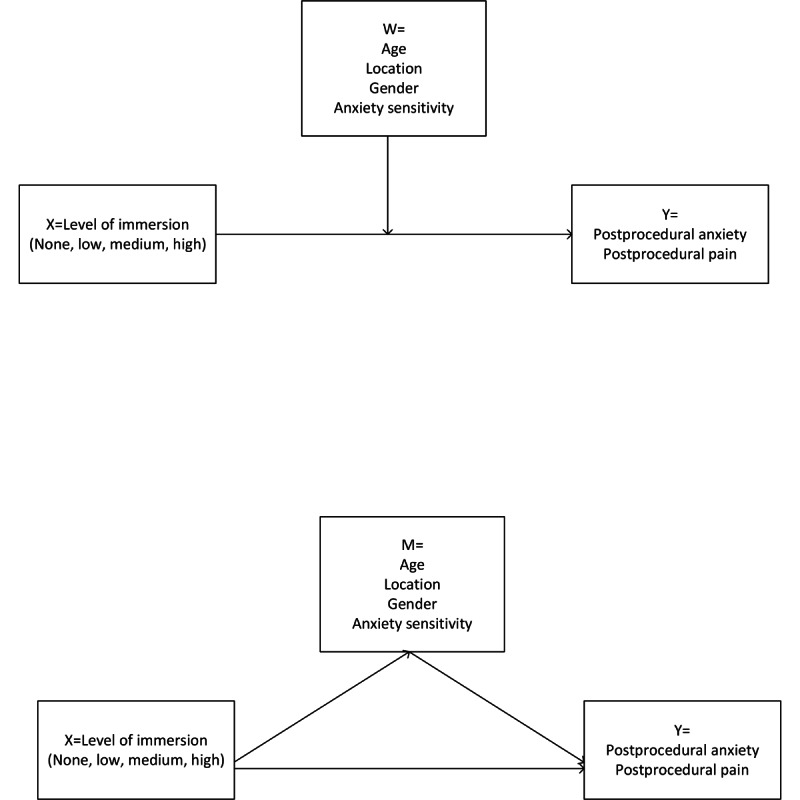
(A) Moderation model. (B) Mediation model.

**Figure 2:**
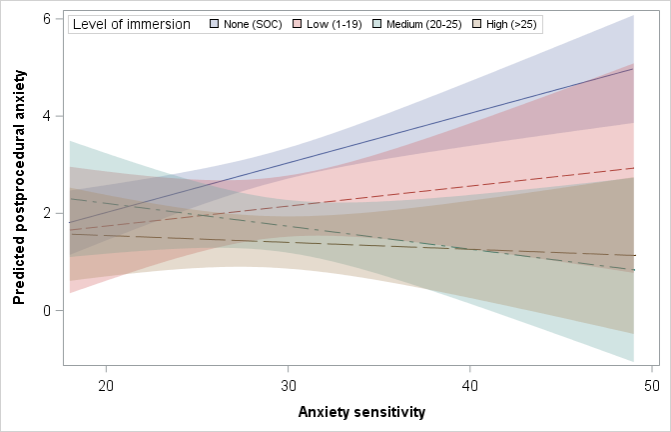
Fit plot with 95% CIs for predicted postprocedural anxiety by anxiety sensitivity grouped by level of immersion. Preprocedural anxiety held constant at 2.107. SOC: standard of care.

Conceptual moderation and mediation models for both postprocedural pain and anxiety are shown in Figure 1. Among the 238 participants in the models, the CASI levels were determined using the 25th, 50th, and 75th percentile of our sample, low CASI (score=24), medium CASI (score=28), and high CASI (score=32). Significant moderation was found between the level of immersion and anxiety sensitivity when predicting postprocedural anxiety (*P*=.01). At a low level of anxiety sensitivity (CASI=24; our sample’s 25th percentile), the no immersion group (SOC) had the highest adjusted mean postprocedural anxiety (2.4, 95% CI 2-2.8), but was not significantly different from the low, medium, or high immersion groups after adjusting for multiple comparisons. At the median level of anxiety sensitivity (CASI=28), significant decreases in postprocedural anxiety were found between high and medium immersion >groups versus no immersion (all *P*<.05; Table 2). These differences were sustained when anxiety sensitivity was high (75th percentile; CASI=32). No significant differences were found when comparing the low, medium, and high levels of immersion to each other (Table 2, Figure 2).

**Table 2:** Postanxiety moderation model between CASI^a^ and level of immersion (N=238)^b^.

CASI level^c^				SOC^d^ (n=123)		Low immersion (n=31)		Medium immersion (n=41)		High immersion (n=43)
**Low CASI (score=24)**										
	LS mean^e^, (95% CI)			2.4 (2.0 to 2.8)		1.9 (1.1 to 2.7)		2.0 (1.3 to 2.8)		1.5 (0.9 to 2.1)
	**Pairwise differences^f^**									
		Versus low immersion	0.5 (–0.7 to 1.7)		N/A^g^		N/A		N/A	
		Versus medium immersion	0.4 (–0.7 to 1.5)		–0.1 (–1.6 to 1.4)		N/A		N/A	
		Versus high immersion	0.9 (–0.1 to 2.0)		0.4 (–1.0 to 1.8)		0.5 (–0.8 to 1.9)		N/A	
**Medium CASI (score=28)**										
	LS mean, (95% CI)			2.8 (2.5 to 3.1)		2.0 (1.4 to 2.7)		1.8 (1.3 to 2.4)		1.4 (0.9 to 2.0)
	**Pairwise differences**									
		Versus low immersion	0.8 (–0.2 to 1.7)		N/A		N/A		N/A	
		Versus medium immersion	1.0 (0.1 to 1.9)^h^		0.2 (–0.9 to 1.4)		N/A		N/A	
		Versus high immersion	1.4 (0.6 to 2.2)^i^		0.6 (–0.5 to 1.8)		0.4 (–0.6 to 1.4)		N/A	
**High CASI (score=32)**										
	LS mean, (95% CI)			3.2 (2.9 to 3.6)		2.2 (1.5 to 2.9)		1.6 (1.0 to 2.2)		1.4 (0.8 to 2.0)
	**Pairwise differences**									
		Versus low immersion	1.0 (–0.05 to 2.1)		N/A		N/A		N/A	
		Versus medium immersion	1.6 (0.7 to 2.5)^i^		0.6 (–0.6 to 1.8)		N/A		N/A	
		Versus high immersion	1.9 (0.9 to 2.8)^i^		0.9 (–0.4 to 2.1)		0.3 (–0.9 to 1.4)		N/A	

^a^CASI: Childhood Anxiety Sensitivity Index.

^b^Resulting from a moderation model controlling for preprocedural anxiety held at the mean of 2.1.

^c^CASI levels were determined using the 25th, 50th, and 75th percentile of our sample.

^d^SOC: standard of care.

^e^LS mean: least squared mean (adjusted mean).

^f^Adjusted pairwise least squared differences with simultaneous 95% CI values. *P* values are adjusted for multiple comparisons using the Bonferroni adjustment.

^g^N/A: not applicable.

^h^*P*<.05.

^i^*P*<.001.

Age, location of procedure, and gender of the patient were not significant moderators of immersion on postprocedural anxiety scores and no significant mediation models were found in our sample. No significant mediation or moderation effects of child characteristics (age, gender, procedure location, and anxiety sensitivity) with level of immersion were found for the outcome of postprocedural pain FPS-R (all *P*>.05).

## Discussion

### Principal Results

The current findings suggest that a higher level of immersion during a VR intervention compared to no immersion decreases postprocedure anxiety among patients undergoing venipuncture procedures. For the purpose of this study, comparisons were made between each level of immersion. Overall, the immersion effect is stronger for patients who reported a higher anxiety sensitivity score, indicating that VR interventions work better to reduce anxiety in individuals who have a higher level of anxiety sensitivity. Patient characteristics such as age, gender, race or ethnicity, significant medical history, location of venipuncture, and type of procedure were not significant moderators of immersion on postprocedural scores, suggesting that VR intervention may be more universal in its application.

### Comparisons With Prior Work

As previously discussed, various studies have concluded that VR reduces patient-reported pain and anxiety during pediatric venipuncture procedures more effectively when compared to the SOC [28-32]; however, this is the first study to analyze patient characteristics and immersion level as predictors of “who benefits” most optimally from the VR intervention.

The current findings support the idea that VR may lend itself to greater benefit for patients undergoing routine painful medical procedures, especially regarding anxiety management. This suggests that VEs need to be highly immersive, especially in the areas of (1) a sense of involvement, (2) perceived realism of the VR game, and (3) a sense of transportation into the experience as measured by the GRIP inventory. As the level of immersion is understood to be a critical element of the VR experience, this study begins to better understand which patients benefit most given their level of immersion. Distraction alone, as is often discussed, may not be the critical element in “why” or “how” VR works, but rather the level or degree of immersion. Previous research has alluded to the fact that deeper levels of engagement or immersion would contribute toward greater VR benefit; however, those ideas were mostly theoretical.

### Future Directions

Over the years, it has been postulated that the greater the number of senses involved in the VR experience, the deeper the sense of immersion. In this study, the VR experience primarily harnessed the patient’s visual and auditory senses, and patients who were highly immersed benefited the most from the intervention. With this finding, it is important to consider the question “Would VR would be more effective if more senses were involved (eg, olfactory, tactile), and whether medium and low levels of immersion would reduce pain and/or anxiety for these patients?”

Future studies investigating unique patient characteristics that drive VR effectiveness may benefit from increasing the number of senses included in their VR experience. VEs designed to engage all 6 senses (vision, hearing, touch, taste, smell, and proprioception) may transform the VR experience beyond what we currently know. Research groups currently engaging in the kinesthetic aspect of VR, olfactory, and tactile vibration could significantly increase the immersion level for all participants, thus enhancing the effects of VR in line with the current findings.

### Limitations

It is important to note that for the purpose of this study, only 1 VE was evaluated (ie, BearBlast), and the current findings are limited to the mentioned procedures and a single virtual experience. However, as the field moves toward a VR pharmacy that allows for choice and customization, the immersive nature may change and be more effective, given personal choice, selection, and preference. Additionally, the participants of this study were primarily Latinx individuals; therefore, it would be beneficial to replicate this study with patients of varying ethnic and racial backgrounds.

### Conclusions

As previously mentioned, factors such as age, gender, and location of venipuncture were not significant. These findings are encouraging as they suggest that the impact of VR is more about the VR experience and less about specific patient and location characteristics. Thus, the use of VR can be implemented in various settings and should be more readily available and accessible for diverse groups of pediatric patients. It was previously discussed that children will remember past painful medical procedures [2-5], which may negatively inform their future perception of potentially painful procedures [7-9]. The use of VR with a high level of immersion can decrease future negative expectations, as well as fear and anxiety about medical procedures. Future studies and interventions should focus on VE and activities with high levels of immersion and interaction in order to best support patient care. Additionally, the use of VR interventions across patient age groups and medical settings may decrease or eliminate the need for pharmacological interventions, the associated negative side-effect profiles, and the negative impact of routine painful medical procedures on patients’ mental health, patients’ medical experiences, and ultimately reduce the fear and anxiety that may impact medical adherence in patients with the critical need for routine and complex chronic medical care.

## Abbreviations

CASI: Childhood Anxiety Sensitivity Index
FPS-R: Faces Pain Scale-Revised
GRIP: Gold-Rizzo Immersion and Presence
PIVC: peripheral intravenous catheter
SOC: standard of care
VAS: visual analog scale
VE: virtual environment
VR: virtual reality

## References

[1] Nordgård R, Låg T (2021). The effects of virtual reality on procedural pain and anxiety in pediatrics: a systematic review and meta-analysis. Front Virtual Real.

[2] Noel M, Chambers CT, McGrath PJ, Klein RM, Stewart SH (2012). The influence of children's pain memories on subsequent pain experience. Pain.

[3] Noel M, Rabbitts JA, Fales J, Chorney J, Palermo TM (2017). The influence of pain memories on children's and adolescents' post-surgical pain experience: a longitudinal dyadic analysis. Health Psychol.

[4] von Baeyer CL, Marche TA, Rocha EM, Salmon K (2004). Children's memory for pain: overview and implications for practice. J Pain.

[5] Gedney JJ, Logan H (2006). Pain related recall predicts future pain report. Pain.

[6] Rocha EM, Marche TA, von Baeyer CL (2009). Anxiety influences children's memory for procedural pain. Pain Res Manag.

[7] Chorney JM, Kain ZN (2009). Behavioral analysis of children's response to induction of anesthesia. Anesth Analg.

[8] El-Housseiny AA, Alamoudi NM, Farsi NM, El Derwi DA (2014). Characteristics of dental fear among Arabic-speaking children: a descriptive study. BMC Oral Health.

[9] Kain ZN, Mayes LC, Caldwell-Andrews AA, Karas DE, McClain BC (2006). Preoperative anxiety, postoperative pain, and behavioral recovery in young children undergoing surgery. Pediatrics.

[10] Birnie KA, Noel M, Parker JA, Chambers CT, Uman LS, Kisely SR, McGrath PJ (2014). Systematic review and meta-analysis of distraction and hypnosis for needle-related pain and distress in children and adolescents. J Pediatr Psychol.

[11] Trottier ED, Doré-Bergeron MJ, Chauvin-Kimoff L, Baerg K, Ali S (2019). Managing pain and distress in children undergoing brief diagnostic and therapeutic procedures. Paediatr Child Health.

[12] (2020). Best practice guidelines for acute pain management in trauma patients. ACS Trauma Quality Programs.

[13] Shiferaw A, Mola S, Gashaw A, Sintayehu A (2022). Evidence-based practical guideline for procedural pain management and sedation for burn pediatrics patients undergoing wound care procedures. Ann Med Surg (Lond).

[14] Stephens RJ, Dettmer MR, Roberts BW, Ablordeppey E, Fowler SA, Kollef MH, Fuller BM (2018). Practice patterns and outcomes associated with early sedation depth in mechanically ventilated patients: a systematic review and meta-analysis. Crit Care Med.

[15] Faber AW, Patterson DR, Bremer M (2013). Repeated use of immersive virtual reality therapy to control pain during wound dressing changes in pediatric and adult burn patients. J Burn Care Res.

[16] Hoffman HG, Patterson DR, Seibel E, Soltani M, Jewett-Leahy L, Sharar SR (2008). Virtual reality pain control during burn wound debridement in the hydrotank. Clin J Pain.

[17] Hoffman HG, Richards TL, Van Oostrom T, Coda BA, Jensen MP, Blough DK, Sharar SR (2007). The analgesic effects of opioids and immersive virtual reality distraction: evidence from subjective and functional brain imaging assessments. Anesth Analg.

[18] Shetty V, Suresh LR, Hegde AM (2019). Effect of virtual reality distraction on pain and anxiety during dental treatment in 5 to 8 year old children. J Clin Pediatr Dent.

[19] Chow H, Hon J, Chua W, Chuan A (2021). Effect of virtual reality therapy in reducing pain and anxiety for cancer-related medical procedures: a systematic narrative review. J Pain Symptom Manage.

[20] Mohammad EB, Ahmad M (2019). Virtual reality as a distraction technique for pain and anxiety among patients with breast cancer: a randomized control trial. Palliat Support Care.

[21] Khadra C, Ballard A, Déry J, Paquin D, Fortin JS, Perreault I, Labbe DR, Hoffman HG, Bouchard S, LeMay S (2018). Projector-based virtual reality dome environment for procedural pain and anxiety in young children with burn injuries: a pilot study. J Pain Res.

[22] Humphrey GB, Boon CMJ, van Linden van den Heuvell GFEC, van de Wiel HBM (1992). The occurrence of high levels of acute behavioral distress in children and adolescents undergoing routine venipunctures. Pediatrics.

[23] Schechter NL, Blankson V, Pachter LM, Sullivan CM, Costa L (1997). The ouchless place: no pain, children's gain. Pediatrics.

[24] Duff AJA (2003). Incorporating psychological approaches into routine paediatric venepuncture. Arch Dis Child.

[25] Cohen LL, MacLaren JE, Lim CS, Steele RG, Elkin TD, Roberts MC (2008). Pain and pain management. Handbook of Evidence-Based Therapies for Children and Adolescents: Bridging Science and Practice.

[26] Slifer KJ (2013). A Clinician's Guide to Helping Children Cope and Cooperate with Medical Care: An Applied Behavioral Approach.

[27] McMurtry CM, Pillai Riddell R, Taddio A, Racine N, Asmundson GJG, Noel M, Chambers CT, Shah V (2015). Far from "just a poke": common painful needle procedures and the development of needle fear. Clin J Pain.

[28] Arane K, Behboudi A, Goldman RD (2017). Virtual reality for pain and anxiety management in children. Can Fam Physician.

[29] Gold JI, Mahrer NE (2018). Is virtual reality ready for prime time in the medical space? A randomized control trial of pediatric virtual reality for acute procedural pain management. J Pediatr Psychol.

[30] Gold JI, SooHoo M, Laikin AM, Lane AS, Klein MJ (2021). Effect of an immersive virtual reality intervention on pain and anxiety associated with peripheral intravenous catheter placement in the pediatric setting: a randomized clinical trial. JAMA Netw Open.

[31] Wong CL, Lui MMW, Choi KC (2019). Effects of immersive virtual reality intervention on pain and anxiety among pediatric patients undergoing venipuncture: a study protocol for a randomized controlled trial. Trials.

[32] Özsoy F, Ulus B (2022). Comparison of two different methods in reducing pain and fear due to dressing change in 7-10 years old children. J Pediatr Res.

[33] Hundert AS, Birnie KA, Abla O, Positano K, Cassiani C, Lloyd S, Tiessen PH, Lalloo C, Jibb LA, Stinson J (2021). A pilot randomized controlled trial of virtual reality distraction to reduce procedural pain during subcutaneous port access in children and adolescents with cancer. Clin J Pain.

[34] İnangil D, Şendir M, Büyükyılmaz F (2020). Efficacy of cartoon viewing devices during phlebotomy in children: a randomized controlled trial. J Perianesth Nurs.

[35] Tennant M, Youssef GJ, McGillivray J, Clark T, McMillan L, McCarthy MC (2020). Exploring the use of immersive virtual reality to enhance psychological well-being in pediatric oncology: a pilot randomized controlled trial. Eur J Oncol Nurs.

[36] Kumari S, Bahuguna R, Garg N, Yeluri R (2021). Immersive and non-immersive virtual reality distraction on pain perception to intraoral injections. J Clin Pediatr Dent.

[37] Miller S, Reid D (2003). Doing play: competency, control, and expression. Cyberpsychol Behav.

[38] Hoffman HG, Sharar SR, Coda B, Everett JJ, Ciol M, Richards T, Patterson DR (2004). Manipulating presence influences the magnitude of virtual reality analgesia. Pain.

[39] Gutierrez-Martinez O, Gutierrez-Maldonado J, Cabas-Hoyos K, Loreto D (2010). The illusion of presence influences VR distraction: effects on cold-pressor pain. Stud Health Technol Inform.

[40] Piskorz J, Czub M (2018). Effectiveness of a virtual reality intervention to minimize pediatric stress and pain intensity during venipuncture. J Spec Pediatr Nurs.

[41] Atzori B, Hoffman HG, Vagnoli L, Patterson DR, Alhalabi W, Messeri A, Lauro Grotto R (2018). Virtual reality analgesia during venipuncture in pediatric patients with onco-hematological diseases. Front Psychol.

[42] Hicks CL, von Baeyer CL, Spafford PA, van Korlaar I, Goodenough B (2001). The Faces Pain Scale-Revised: toward a common metric in pediatric pain measurement. Pain.

[43] Klimek L, Bergmann KC, Biedermann T, Bousquet J, Hellings P, Jung K, Merk H, Olze H, Schlenter W, Stock P, Ring J, Wagenmann M, Wehrmann W, Mösges R, Pfaar O (2017). Visual analogue scales (VAS): measuring instruments for the documentation of symptoms and therapy monitoring in cases of allergic rhinitis in everyday health care. Allergo J Int.

[44] Silverman WK, Fleisig W, Rabian B, Peterson RA (1991). Childhood Anxiety Sensitivity Index. J Clin Child Psychol.

[45] Hayes AF (2022). Introduction to Mediation, Moderation, and Conditional Process Analysis: A Regression-Based Approach, Third Edition.

